# Pap12-6: A host defense peptide with potent immunomodulatory activity in a chicken hepatic cell culture

**DOI:** 10.1371/journal.pone.0302913

**Published:** 2024-05-10

**Authors:** Rege Anna Márton, Csilla Sebők, Máté Mackei, Patrik Tráj, Júlia Vörösházi, Ágnes Kemény, Zsuzsanna Neogrády, Gábor Mátis

**Affiliations:** 1 Division of Biochemistry, Department of Physiology and Biochemistry, University of Veterinary Medicine, Budapest, Hungary; 2 National Laboratory of Infectious Animal Diseases, Antimicrobial Resistance, Veterinary Public Health and Food Chain Safety, University of Veterinary Medicine, Budapest, Hungary; 3 Department of Pharmacology and Pharmacotherapy, Medical School, University of Pécs, Pécs, Hungary; 4 Department of Medical Biology, Medical School, University of Pécs, Pécs, Hungary; Xinyang Normal University, CHINA

## Abstract

In the fight against antimicrobial resistance, host defense peptides (HDPs) are increasingly referred to as promising molecules for the design of new antimicrobial agents. In terms of their future clinical use, particularly small, synthetic HDPs offer several advantages, based on which their application as feed additives has aroused great interest in the poultry sector. However, given their complex mechanism of action and the limited data about the cellular effects in production animals, their investigation is of great importance in these species. The present study aimed to examine the immunomodulatory activity of the synthetic HDP Pap12-6 (PAP) solely and in inflammatory environments evoked by lipoteichoic acid (LTA) and polyinosinic-polycytidylic acid (Poly I:C), in a primary chicken hepatocyte–non-parenchymal cell co-culture. Based on the investigation of the extracellular lactate dehydrogenase (LDH) activity, PAP seemed to exert no cytotoxicity on hepatic cells, suggesting its safe application. Moreover, PAP was able to influence the immune response, reflected by the decreased production of interleukin (IL)-6, IL-8, and “regulated on activation, normal T cell expressed and secreted”(RANTES), as well as the reduced IL-6/IL-10 ratio in Poly I:C-induced inflammation. PAP also diminished the levels of extracellular H_2_O_2_ and nuclear factor erythroid 2-related factor 2 (Nrf2) when applied together with Poly I:C and in both inflammatory conditions, respectively. Consequently, PAP appeared to display potent immunomodulatory activity, preferring to act towards the cellular anti-inflammatory and antioxidant processes. These findings confirm that PAP might be a promising alternative for designing novel antimicrobial immunomodulatory agents for chickens, thereby contributing to the reduction of the use of conventional antibiotics.

## Introduction

Over the past decades, the increased consumption of meat has prompted the maximization of production in the livestock sector [1, 2]. To fulfill this demand, one of the most common methods was the excessive use of conventional antibiotics (ABs), which can be considered a major contributor to the spread of antimicrobial resistance (AMR) [1]. Besides being a serious concern in the field of veterinary medicine due to treatment failures, AMR poses a severe threat to global public health as well [1]. Therefore, the need to search for novel candidates with antimicrobial activity has been increasingly highlighted [3]. In this context, host defense peptides (HDPs)–also known as antimicrobial peptides (AMPs)–are particularly studied and promising molecules, thereby reducing the use of ABs, and promoting the fight against AMR [3].

HDPs are small, usually less than 50 amino acid residues containing, cationic peptides [4], naturally produced in every living organism as crucial components of their innate immune system [5]. Although they first attracted attention for their broad-spectrum direct antimicrobial activity, in recent years, great importance has been attached to their immunomodulatory effects which, in contrast to the former, are more likely to be observed in *in vivo* or *in vivo*-like conditions as well [6]. Immunomodulation by HDPs can be exerted in a variety of ways, such as promoting the recruitment of leukocytes, stimulating chemotaxis, inducing the production of pro- and anti-inflammatory cytokines, supporting the activation or differentiation of immune cells, and neutralizing endotoxins [7]. Hence, their mode of action is pleiotropic and shows high complexity [8]; therefore, it may depend on specific biological circumstances [7]. Based on these characteristics, in contrast to the ABs, HDPs offer a multiple and versatile antimicrobial nature, which makes them suitable for designing new antimicrobial agents [2].

However, due to their peptide nature, there are still some drawbacks that need to be addressed for future clinical use [9]. Among these disadvantages are the potential toxicity to the host cells, the high susceptibility to protease enzymes and hence, their questionable pharmacokinetics, as well as high production costs on a large scale [9–11]. There are various approaches to counteract these obstacles, one of which is the design and application of small, synthetic peptides [12]. Compared to naturally occurring HDPs, they offer several advantages, such as easier and less expensive production, lower immunogenicity, and the possibility to be tuned according to the desired stability, half-life, or specificity [12]. Based on these, the application of small, synthetic HDPs as feed additives aroused great interest in the poultry sector, as they are suggested to provide various benefits, including immunomodulation, inhibition of the spread of foodborne pathogens, minimization of carcass contamination, reduction of the prevalence of AMR [13], or growth-promoting effects [3].

Pap12-6 (PAP) is a 12-meric synthetic HDP, derived from the N-terminal end of the natural papiliocin found in swallowtail butterfly (*Papilio xuthus*) larvae [14]. Even though its parent peptide was described to exert potent antibacterial and anti-inflammatory activity [15, 16], the length of papiliocin and the resulting above-mentioned drawbacks prompted the researchers to design shorter derivatives while maintaining or even enhancing its beneficial effects. Among them, the development of PAP contributed to a significantly shorter peptide with high host cell selectivity, broad-spectrum antibacterial effect, and efficient anti-inflammatory activity [14]. From the latter aspect, PAP reduced the production of pro-inflammatory cytokines, such as interleukin (IL)-6, IL-1α, and tumor necrosis factor (TNF)-α in different cell cultures exposed to inflammatory stimuli [14, 17]. Moreover, in septic mouse models, it was able to improve survival, relieve symptoms, decrease the level of pro-inflammatory cytokines in serum and different organs, prevent the infiltration by neutrophils, and as a consequence, protect the host from inflammation [14, 17].

Despite the promising results, only a few studies have been implemented to investigate the immunomodulatory effects of PAP; therefore, additional research is needed to fully elucidate its mechanism of action. For example, to the best of the authors’ knowledge, neither PAP nor any other derivatives of papiliocin were investigated in hepatic cells. However, the liver plays a key role in regulating inflammatory processes as it is constantly and directly exposed to harmless as well as harmful antigenic load and metabolites originating from the gastrointestinal tract [18]. This requires a highly orchestrated local immune system, coordinated by liver innate cells, such as monocyte-derived macrophages, resident macrophages (also known as Kupffer-cells [KCs]), or dendritic cells (DCs), and various antimicrobial components like inflammatory cytokines and chemokines, acute phase proteins, or the complement system [19]. Briefly, upon microbial stimulation, the pattern recognition receptors (PRRs)–such as Toll-like receptors (TLRs)–of KCs and DCs detect the pathogen-associated molecular patterns (PAMPs) [19]. Subsequently, PRR activation induces downstream signaling that leads to an inflammatory response [19], characterized by the production of pro-inflammatory cytokines and chemokines, the recruitment of neutrophils and monocytes, and the formation of reactive oxygen species (ROS) [20].

Another limitation of the currently available research data about papiliocins is that the different, previously tested derivatives were investigated only in mouse- or human-derived cell lines. However, as production animals, like chickens, are particularly challenged by pathogens and the presence of AMR [21], the examination of HDPs at their cellular level is of great importance. Therefore, the goal of the present study was to investigate the putative immunomodulatory effects of PAP in a primary hepatocyte–non-parenchymal cell co-culture of chicken origin. To evoke inflammation, lipoteichoic acid (LTA), as a TLR2-agonist from the cell wall of Gram-positive bacteria [22], and polyinosinic-polycytidylic acid (Poly I:C), a TLR3-agonist synthetic double-stranded RNA (dsRNA) analog [23] were used.

## Materials and methods

### Isolation of the cells

For the isolation of the cells, a 3-week-old male Ross-308 broiler chicken was used, which was kept in the animal house of the Department of Physiology and Biochemistry, University of Veterinary Medicine Budapest, Hungary. Water was provided *ad libitum*, the chicken was fed according to the instructions of the breeder, and all efforts were taken to maintain animal well-being and health. The present experiment was in line with the European Union’s laws, approved by the Local Animal Welfare Committee, and allowed by the Government Office (permission number: GK-419/2020; date of approval: 11 May 2020). Unless stated by the authors otherwise, the compounds and chemicals described below were purchased from Merck KGaA (Darmstadt, Germany).

Isolation of the cells was carried out according to the protocol of our research group [24]. After decapitation under CO_2_ narcosis, removing the abdominal feathers, and disinfecting the skin of the abdominal region, the body cavity was opened. Next, the *gastropancreaticoduodenal* vein was cannulated with a 22G-size venous cannula, and a three-step perfusion of the liver was performed at a flow rate of 30 ml/min, using freshly preheated (40°C) and oxygenated (Carbogen, 95% O_2_; 5% CO_2_; flow rate of 1 l/min) solutions. To begin with, 150 ml of Hanks’s Balanced Salt Solution (HBSS) buffer supplemented with ethylene glycol-bis(2-aminoethyl ether)-N,N,N’,N’-tetraacetic acid (EGTA) was used, followed by flushing with 150 ml EGTA-free HBSS buffer. The third step was carried out by the application of 100 ml HBSS solution, containing 100 mg type IV collagenase, 7 mM CaCl_2,_ and 7 mM MgCl_2_. After removing the liver and the Glisson’s capsule, cells were suspended in 50 ml bovine serum albumin (BSA, 2.5%)-containing HBSS buffer, and filtered through a three-layer sterile gauze sheet. As a next step, the resulting suspension of the cells was incubated for 45 minutes on ice. Thereafter, centrifugation (3 minutes, 100 x g) of the cell suspension was performed three times, collecting each supernatant separately, and resuspending the resulting sediment in Williams’ Medium E (supplemented with 5% fetal bovine serum [FBS], 0.22% NaHCO_3_, 2 mM glutamine, 50 mg/ml gentamycin, 4 g/l dexamethasone, 20 IU/l insulin and 0.5 g/ml amphotericin B) at every step, thereby gaining a hepatocyte-containing cell suspension after the third centrifugation. On the other hand, the supernatants collected were mixed and spun (10 minutes, 350 x g), and centrifugation (10 minutes, 800 x g) was anew performed with the resulting supernatant. Thereafter, the pellet was resuspended in Williams’ Medium E, thereby collecting a suspension rich in non-parenchymal cells. Hepatocytes and macrophages, respectively, were previously characterized in the two fractions by immunofluorescent staining and flow cytometry [24]. In the above, former experiment of our research group, chicken-specific, fluorescein isothiocyanate (FITC)-coupled anti-albumin was used to detect the isolated and cultured hepatocytes, whereas macrophages in the non-parenchymal cell-rich fraction were labeled by chicken macrophage-specific phycoerythrin (PE)-conjugated antibodies. The isolation of the cells was conducted the same way in the present study, ensuring the presence of the same types of cells in the corresponding fractions. To confirm this, the morphology of the isolated cells and that of confluent cell cultures was checked after Giemsa staining. To assess cellular viability before seeding, a trypan blue exclusion test was carried out in Bürker’s chambers for both cell suspensions. Prior to seeding, the two fractions were diluted according to the cell count, and the hepatocyte-containing suspension was blended with the non-parenchymal cell-rich fraction in a 6 to 1 ratio, receiving a total concentration of 10^6^ cells/ml. Cells were seeded in a volume of 400 μl suspension/well into 24-well cell culture plates (Greiner Bio-One Hungary Kft., Mosonmagyaróvár, Hungary) previously coated with type I rat tail collagen (10 μg / cm^2^). After 4 hours of incubation at 37°C and 5% CO2, the cell culture media were changed, and the cells were incubated again for 24 hours under the same circumstances.

### Treatments

Chicken hepatocyte–non-parenchymal cell co-cultures were treated according to Table 1., using the previously mentioned supplemented Williams’ Medium E culture media, however, without the use of FBS. Two different inflammatory conditions were evoked by the addition of LTA (50 μg/ml) or Poly I:C (50 μg/ml). PAP (Isca Biochemicals Ltd., Exeter, UK) was applied in three different concentrations (5, 25, and 50 μg/ml) solely and together with LTA and Poly I:C respectively. Cells receiving only Williams’ Medium E were considered as Control. After 24 hours of treatment, cell culture media samples were taken and frozen at -80°C until further measurements. Thereafter, to gain lysate samples, culture plates were washed first with 300 μl/well of phosphate-buffered saline (PBS) solution, followed by the addition of 50 μl/well of M-PER^™^ Mammalian Protein Extraction Reagent (Thermo Fisher Scientific Inc., Waltham, MA, USA). Thereafter, the cells were scraped from the bottom of the wells and frozen to -80°C until further processing.

**Table 1:** Treatment groups applied on primary hepatocyte–non-parenchymal cell co-cultures of chicken origin.

Treatment group	PAP	LTA	Poly I: C
**Control**	—	—	—
**PAP-1**	5 μg/ml	—	—
**PAP-2**	25 μg/ml	—	—
**PAP-3**	50 μg/ml	—	—
**LTA**	—	50 μg/ml	—
**LTA+PAP-1**	5 μg/ml	50 μg/ml	—
**LTA+PAP-2**	25 μg/ml	50 μg/ml	—
**LTA+PAP-3**	50 μg/ml	50 μg/ml	—
**PI:C**	—	—	50 μg/ml
**PI:C+PAP-1**	5 μg/ml	—	50 μg/ml
**PI:C+PAP-2**	25 μg/ml	—	50 μg/ml
**PI:C+PAP-3**	50 μg/ml	—	50 μg/ml

PAP-1-3 = different concentrations of Pap12-6 (PAP); LTA = addition of 50 μg/ml lipoteichoic acid (LTA); PI:C = addition of 50 μg/ml polyinosinic-polycytidylic acid (Poly I:C).

### Measurements

#### Cellular viability

For the investigation of the cellular viability, the colorimetric Lactate Dehydrogenase Activity Assay Kit was utilized. In case of membrane damage, the cells release lactate dehydrogenase (LDH) into the culture media, leading to the production of NADH^+^, the amount of which can be specifically detected. According to the manufacturer’s instructions, 50 μl of culture media samples were applied on a 96-well microplate, followed by the addition of 50 μl of NAD^+^-containing freshly prepared Master Reaction Mix. Absorbance values were measured with a Multiscan GO 3.2 reader (Thermo Fisher Scientific Inc., Waltham, MA, USA) at 450 nm, after 2 minutes of incubation at 37°C, protected from light. Readings were continued every 5 minutes until the absorbance of the most active sample became higher than the value of the most concentrated standard.

#### Inflammatory markers

To investigate the influence of PAP on the immune response, the levels of IL-6, IL-8, IL-10, interferon (IFN)-γ and regulated upon activation, normal T cell expressed and secreted (RANTES) were measured in the cell culture media. IL-8 was examined with chicken-specific ELISA (MyBioSource Inc., San Diego, CA, USA, Cat. Nr.: MBS289628), using a sandwich technique, following the manufacturer’s instructions. Absorbance values were measured with a Multiscan GO 3.2 reader, at 450 nm.

The concentrations of IL-6, IL-10, IFN-γ, and RANTES were determined by Luminex xMAP Technology, using Milliplex Chicken Cytokine/Chemokine Panel 1 –Immunology Multiplex Assay (Cat. Nr.: GCYT1-16K). A 96-well microplate belonging to the kit was filled with duplicates of 25 μl culture media samples. Thereafter, each well was loaded with 25 μl of four sets of colored capture antibody-coated beads, followed by overnight incubation and washing. As a next step, biotinylated detection antibody and streptavidin phycoerythrin solutions were applied. Thereupon, the plate was treated with 150 ml of drive fluid, and the beads were resuspended for 5 minutes on a plate shaker. As a last step, reading was executed using a Luminex MAGPIX^®^ instrument, and data were assembled by the Luminex xPonent 4.2 program. According to the median fluorescence intensity of the beads, standard curves were generated by Belysa Immunoassay Curve Fitting software for all analytes.

#### Redox markers

For the examination of the oxidative state, the levels of extracellular (EC) H_2_O_2_ and nuclear factor erythroid 2-related factor 2 (Nrf2) (also known as nuclear factor erythroid-derived 2-like 2 [NFE2L2]) were assayed. The EC H_2_O_2_ concentrations were determined using the fluorometric Amplex Red method (Thermo Fisher Scientific, Waltham, MA, USA), according to the instructions of the manufacturer. A 96-well microplate was loaded with 50 μl of culture media samples, supplemented with 50 μl of prior-to-use prepared Amplex Red Working Solution, containing Amplex Red Stock Solution, HRP Stock Solution, and 1X Reaction Buffer. After incubating for 30 minutes at room temperature (24°C), protected from light, a Victor X2 2030 fluorometer (Perkin Elmer Inc., Waltham, MA, USA) was used to read the fluorescence values at 530 nm (excitation) and 590 nm (emission).

Levels of Nrf2 were assayed using a Chicken NFE2L2 (Nuclear Factor, Erythroid Derived 2 Like Protein 2) ELISA Kit (MyBioSource Inc., San Diego, CA, USA, Cat. Nr.: MBS8807992), following the protocol provided by the manufacturer. Absorbance values were determined with a Multiscan GO 3.2 reader, at 450 nm.

Total protein concentrations were determined by utilizing the Pierce^™^ Bicinchoninic Acid (BCA) Protein Assay (Thermo Fisher Scientific, Waltham, MA, USA, Cat. Nr.:23227), using BSA as standard. According to the manufacturer’s instructions, 25 μl of cell lysate samples were added to a 96-well microplate, followed by the administration of 200 μl freshly prepared Reagent A+B Solution. After shaking for 30 seconds, the plate was incubated for 30 minutes at 37°C, protected from light. Absorbance values were measured with a Multiscan GO 3.2 reader, at 562 nm. Total protein values were used to standardize the results of each cellular measurement.

### Statistical analysis

Statistical analysis of data was performed by using R v. 4.0.3 (R Core Team, 2020). Based on Shapiro-Wilk tests, data from treatment groups showed non-normal distribution; therefore, the Wilcoxon signed-rank test was utilized for pairwise comparisons. The difference was considered significant if the resulting p-value was lower than 0.05. Treatment groups solely receiving PAP, LTA, or Poly I:C were compared to Control, whereas the cells treated with the combinations of PAP and LTA, as well as PAP and Poly I:C were compared to groups exposed to only LTA and Poly, respectively. Correlations were examined by MetaboAnalyst 5.0 software, using Pearson’s correlation test. According to Mukaka, 2009 [25], correlations were described as “very high”, “high”, “moderate”, “low” or “negligible”, based on the correlation coefficients (r) being ±0.90–1.00, ±0.70–0.90, ±0.50–0.70, ±0.30–0.50 and 0.00-±0.30, respectively. Graphs were created by using Prism 9 (GraphPad Software Inc., San Diego, CA, V 9.2.1). Heat map of correlations was prepared by PyCharm v. 2023.3.4. (JetBrains s.r.o, Prague, Czech Republic).

## Results

### Cellular viability

**Fig 1:**
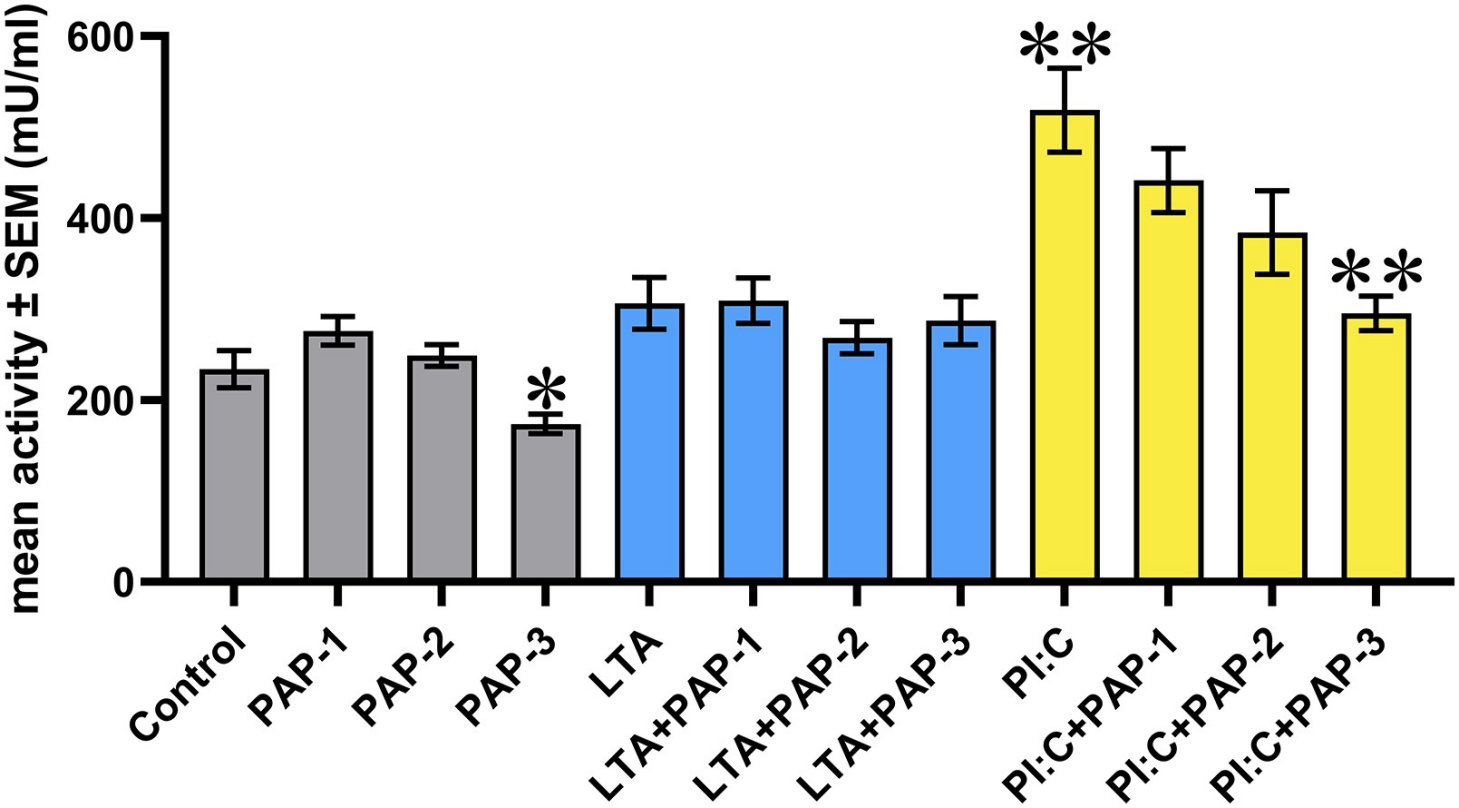
Bar graph showing extracellular lactate dehydrogenase (LDH) activity. Chicken hepatocyte–non-parenchymal cell co-cultures were treated with three different concentrations of Pap12-6 (PAP) solely and in combination with lipoteichoic acid (LTA) or polyinosinic-polycytidylic acid (Poly I:C). Grey color refers to treatment groups without the addition of LTA or Poly I:C, whereas blue color refers to treatment with LTA, and yellow color refers to treatment with Poly I:C. Columns represent means ± SEM (n = 6 / treatment group). PAP-1 = 5 μg/ml PAP, PAP-2 = 25 μg/ml PAP, PAP-3 = 50 μg/ml PAP, LTA = 50 μg/ml LTA, PI:C = 50 μg/ml Poly I:C. Cells receiving none of the treatments are considered as Control. Groups PAP-1, PAP-2, PAP-3, LTA and PI:C were compared to Control. Combinations of LTA and PAP (LTA+PAP-1, LTA+PAP-2 and LTA+PAP-3) were compared to the group LTA. Combinations of Poly I:C and PAP (PI:C+PAP-1, PI:C+PAP-2 and PI:C+PAP-3) were compared to the group PI:C. Asterisks indicate significant differences between the above-mentioned treatment groups. *p < 0.05, **p < 0.01.

With regard to the EC LDH activity, the solely applied highest concentration of PAP (50 μg/ml) contributed to a significant decrease (p = 0.026). In addition, Poly I:C-evoked inflammation led to a significant elevation (p = 0.002), which was significantly reduced by PAP at its concentration of 50 μg/ml (p = 0.002) (Fig 1).

### Inflammatory markers

**Fig 2:**
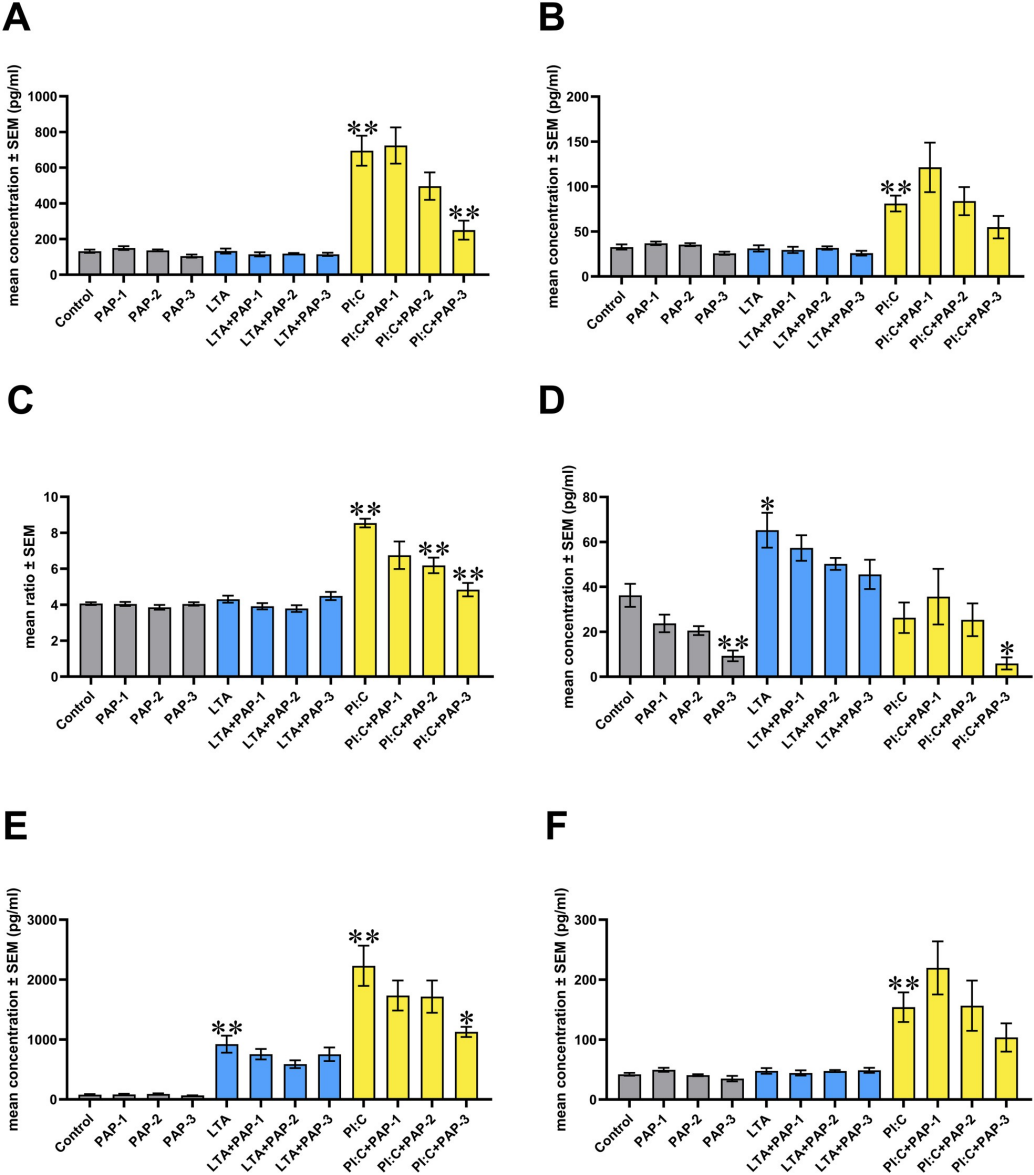
Bar graphs showing the concentrations of different inflammatory markers examined. (A) IL-6 levels. (B) IL-10 levels. (C) IL-6/IL-10 ratio. (D) IL-8 levels. (E) RANTES levels. (F) IFN-γ levels. Chicken hepatocyte–non-parenchymal cell co-cultures were treated with three different concentrations of Pap12-6 (PAP) solely and in combination with lipoteichoic acid (LTA) or polyinosinic-polycytidylic acid (Poly I:C). Grey color refers to treatment groups without the addition of LTA or Poly I:C, whereas blue color refers to treatment with LTA, and yellow color refers to treatment with Poly I:C. Columns represent means ± SEM (n = 6 / treatment group). PAP-1 = 5 μg/ml PAP, PAP-2 = 25 μg/ml PAP, PAP-3 = 50 μg/ml PAP, LTA = 50 μg/ml LTA, PI:C = 50 μg/ml Poly I:C. Cells receiving none of the treatments are considered as Control. Groups PAP-1, PAP-2, PAP-3, LTA and PI:C were compared to Control. Combinations of LTA and PAP (LTA+PAP-1, LTA+PAP-2 and LTA+PAP-3) were compared to the group LTA. Combinations of Poly I:C and PAP (PI:C+PAP-1, PI:C+PAP-2 and PI:C+PAP-3) were compared to the group PI:C. Asterisks indicate significant differences between the above-mentioned treatment groups.*p < 0.05, **p < 0.01.

When measuring IL-6 concentrations, Poly I:C was found to exert a significant increasing effect (p = 0.002), which was significantly reduced by the concomitant application of PAP at its 50 μg/ml concentration (p = 0.004) (Fig 2A).

In the case of IL-10, the level of the cytokine was changed only by the sole Poly I:C exposure, causing a significant increase (p = 0.002) (Fig 2B).

IL-6/IL-10 ratio was found to be heightened in the case of inflammation evoked by Poly I:C (p = 0.002), which elevation was decreased by the addition of PAP at its concentrations of 25 and 50 μg/ml (p = 0.002 in both cases) (Fig 2C).

In the case of IL-8 (syn. CXCLi2 in chickens), the levels were found to decrease by the sole and Poly I:C-combined application of PAP at its 50 μg/ml concentration (p = 0.004 and p = 0.030, respectively), whereas LTA exerted a significant increasing effect (p = 0.026) (Fig 2D).

Concentrations of RANTES were elevated by both LTA (p = 0.002) and Poly I:C (p = 0.002), the latter of which was significantly decreased by the highest concentration (50 μg/ml) of PAP (p = 0.015) (Fig 2E).

Regarding the production of IFN-γ, the only change observed was the significant elevation caused by Poly I:C (p = 0.002) (Fig 2F).

### Redox markers

**Fig 3:**
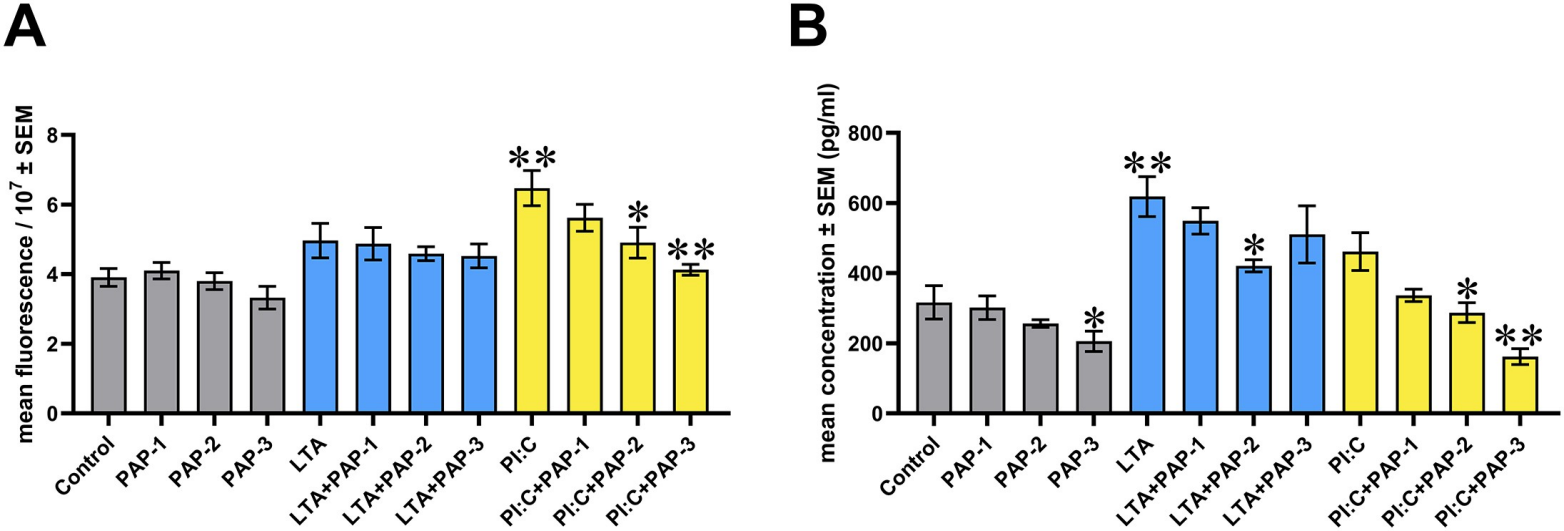
Bar graphs showing the concentrations of different redox markers examined. (A) EC H_2_O_2_ levels. (B) Nrf2 levels. Chicken hepatocyte–non-parenchymal cell co-cultures were treated with three different concentrations of Pap12-6 (PAP) solely and in combination with lipoteichoic acid (LTA) or polyinosinic-polycytidylic acid (Poly I:C). Grey color refers to treatment groups without the addition of LTA or Poly I:C, whereas blue color refers to treatment with LTA, and yellow color refers to treatment with Poly I:C. Columns represent means ± SEM (n = 6 / treatment group). PAP-1 = 5 μg/ml PAP, PAP-2 = 25 μg/ml PAP, PAP-3 = 50 μg/ml PAP, LTA = 50 μg/ml LTA, PI:C = 50 μg/ml Poly I:C. Cells receiving none of the treatments are considered as Control. The treatment groups were compared to one another using Wilcoxon sign-ranked tests. Groups PAP-1, PAP-2, PAP-3, LTA and PI:C were compared to Control. Combinations of LTA and PAP (LTA+PAP-1, LTA+PAP-2 and LTA+PAP-3) were compared to the group LTA. Combinations of Poly I:C and PAP (PI:C+PAP-1, PI:C+PAP-2 and PI:C+PAP-3) were compared to the group PI:C. Asterisks indicate significant differences between the above-mentioned treatment groups. *p < 0.05, **p < 0.01.

When measuring EC H_2_O_2_ levels, Poly I:C alone contributed to a significant increase (p = 0.002), which was lessened by the addition of PAP at its 25 and 50 μg/ml concentrations (p = 0.041 and p = 0.002, respectively) (Fig 3A).

In the case of Nrf2, PAP at 50 μg/ml alone resulted in a significantly decreased level (p = 0.015). Furthermore, LTA was found to exert a significant elevating effect (p = 0.009), whereas the change caused by LTA was significantly reduced by PAP treatment at its 25 μg/ml concentration (p = 0.015). In addition, at its concentrations of 25 and 50 μg/ml, PAP was able to reduce the Nrf2 level, when applied together with Poly I:C (p = 0.015 and p = 0.002, respectively) (Fig 3B).

### Correlations

**Fig 4:**
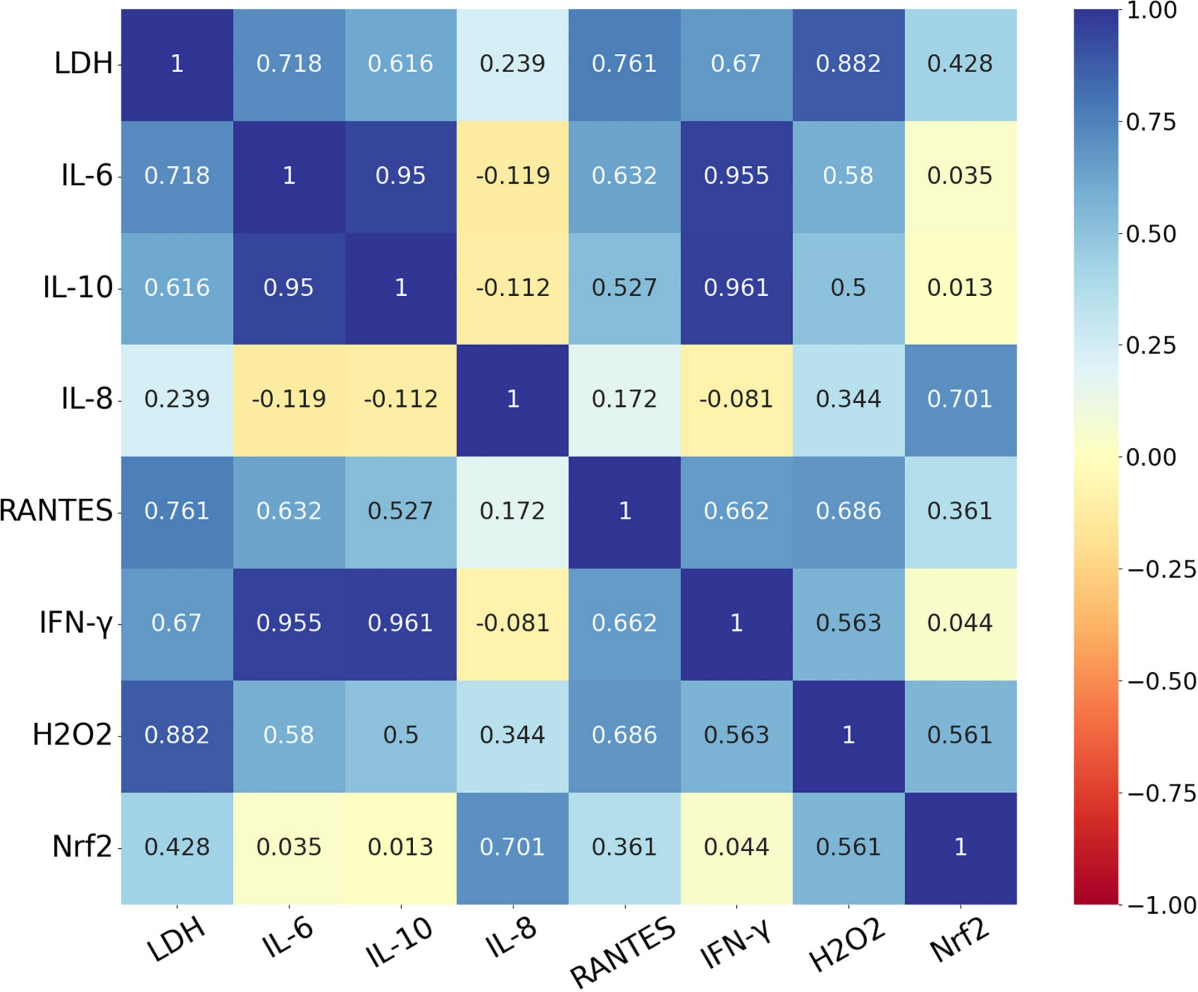
Heat map showing the correlations between different parameters with the respective correlation coefficient (r) values. Blue color refers to positive, whereas red color refers to negative correlation. LDH = lactate dehydrogenase, IL-6 = interleukin-6, IL-10 = interleukin-10, IL-8 = interleukin 8, RANTES = regulated on activation, normal T cell expressed and secreted, IFN-γ = interferon-γ, H2O2 = hydrogen peroxide, Nrf2 = nuclear factor erythroid 2-related factor 2.

Regarding the correlations, very high positive correlations were found between IFN-γ and IL-10 (r = 0.961; p<0.001), IFN-γ and IL-6 (r = 0.951; p<0.001), as well as IL-6 and IL-10 (r = 0.950; p<0.001) (Fig 4). High positive correlations were observed between LDH and each of EC H_2_O_2_ (r = 0.882; p<0.001), RANTES (r = 0.761; p<0.001), and IL-6 (r = 0.712; p<0.001), as well as between IL-8 and Nrf2 (r = 0.701; p<0.001). In addition, moderate but significant positive correlations were revealed between EC H_2_O_2_ and each of IL-6 (r = 0.580, p<0.001), IL-10 (r = 0.500; p<0.001), IFN-γ (r = 0.563, p<0.001), RANTES (r = 0.686; p<0.001), and Nrf2 (r = 0.561, p<0.001). Moreover, moderate and significant positive correlations were found between LDH and each of IFN-γ (r = 0.670, p<0.001) and IL-10 (r = 0.616, p<0.001), as well as between RANTES and each of IL-10 (r = 0.527; p<0.001), IL-6 (r = 0.632; p<0.001), and IFN-γ (r = 0.662; p<0.001). Detailed results of correlations can be found in Fig 4 and S1 Table.

## Discussion

The widespread occurrence of AMR poses a serious risk to the control of infectious diseases in veterinary and human medicine. In livestock farming, the reduction of the use of ABs, and meanwhile, the maintenance of animal welfare, health, and production efficiency requires an urgent search for new antimicrobial agents [26]. In this field, HDPs are considered outstanding candidates as they provide various advantageous features besides their broad-spectrum antimicrobial activity, such as their potent immunomodulatory effects [27]. Therefore, they aroused great interest from the poultry sector, and there have already been studies revealing the beneficial impact of certain HDPs on the chicken immune response in *in vitro* [28–31] and *in vivo* conditions [32–37]. However, the capability of HDPs to modulate immunological processes is not universal and greatly affected by certain biological environment, such as the cell types, signaling pathways and receptors involved, the current inflammatory stimulus, or the concentration of the peptide [7]. For this reason, and to better predict the *in vivo* effects of HDPs, the examination of their mechanism of action at a cellular level is of great importance [11].

The present study aimed to investigate the putative immunomodulatory activity of PAP in a chicken primary hepatocyte–non-parenchymal cell co-culture solely, and in different inflammatory conditions evoked by LTA and Poly I:C. Both of the applied TLR-agonists contribute to the activation of transcription factor nuclear factor-κB (NF-κB), and subsequently, the production of pro-inflammatory mediators; however, it is achieved via different downstream signaling pathways. While LTA-induced triggering of TLR2 recruits myeloid differentiation primary response 88 protein (MyD88) [38–40], the stimulation of TLR3 by Poly I:C leads to the recruitment of Toll-interleukin-1 receptor (TIR)‍-domain-containing adaptor-inducing interferon-‍β (TRIF) [41]. The activation of NF-κB is an evolutionary conserved defense mechanism against infection; however, its exaggerated stimulation can cause detrimental consequences to production animals, such as the impairment of their reproductive and production efficiency and the risk of developing chronic inflammatory conditions [42]. NF-κB also has a crucial role in reacting to oxidative stress which is increasingly suggested to be tightly connected to the inflammatory state [43]. Since the presence and significance of both TLR3 and TLR2 have been identified in chickens [40], and the regulatory role of NF-κB is also suggested in them [42], LTA and Poly I:C are potent agents to induce inflammation in poultry cells and have already been applied by our research group successfully in former studies for this purpose [30, 31, 44, 45]. In the present study, Poly I:C caused a significant increase in the production of IL-6, IFN-γ, RANTES, and EC H_2_O_2_, and it contributed to a higher IL-6/IL-10 ratio, suggesting the induced inflammatory state. In addition, reflected by the increased levels of RANTES and IL-8, LTA also evoked inflammation in the cell cultures.

### Cellular viability

In order to enable the future use of HDPs, the examination of their possible toxicity towards eukaryotic cells is of great importance [46]. However, most of the studies assaying their cytotoxic effects were conducted on mammalian red blood cells or cancerous cell lines [47], and far fewer data are available about the impact of HDPs on the viability of poultry cells. In the present study, the EC LDH activity was determined to assess the integrity changes in the cell membrane and hence, the cellular viability. According to our results, none of the administered peptide concentrations displayed cytotoxic effects. Moreover, the highest concentration of PAP was able to enhance the cellular membrane integrity when applied alone and restore the membrane damage caused by Poly I:C. Due to the high positive correlations found between LDH and each of the pro-inflammatory RANTES and IL-6, the membrane damage caused by Poly I:C is hypothesized to befall primarily through pyroptosis, the mechanism of which results in transcellular pore formation [45]. This inflammatory cell death, leading to the fast leakage of LDH and inflammatory mediators, has already been suggested by our research group to occur after Poly I:C-treatment of the same type of cell culture [45], and demonstrated by Lian et al., 2012 to be elicited in human neonatal primary keratinocytes [48]. The observed effects of PAP on cellular viability are in agreement with previous reports, as the peptide was found to exert no cytotoxic effects in RAW 264.7 mouse macrophage cell line, HaCaT human keratinocyte cell line [14, 17], and HEK-293 human embryonic kidney cell line [14]. Based on these findings, the administration of PAP on eukaryotic cells is suggested to be harmless. Moreover, it could even improve the viability of the hepatic cells in Poly I:C-induced inflammation.

### Inflammatory markers

To investigate the putative effects of PAP on the immune response, the levels of IL-6, IL-8, IL-10, RANTES, and IFN-γ were determined. In the liver, IL-6 is synthesized mainly by KCs upon stimulation of TLRs or tissue injury, providing the induction of acute phase response [49] and hence, early protection against infection [50]. Under physiological circumstances, IL-6 has a crucial role in maintaining the liver defense mechanisms and homeostasis; however, its exaggerated activation can contribute to harmful consequences [49, 50]. In our study, PAP was able to decrease the Poly I:C-induced increase in IL-6 production and the IL-6/IL-10 ratio at its highest concentration, thereby alleviating inflammation. It is in line with former findings, as PAP was able to restore the LPS-induced elevation of IL-6 concentrations in RAW 264.7 mouse macrophage cell lines [14, 17]. Under *in vivo* conditions, serum IL-6 levels of *Escherichia coli*-challenged mice were also diminished by the administration of PAP [14, 17]. Moreover, native papiliocin [16] and its other derivatives [17, 51, 52] were also found to exert the same effect in different cell cultures, suggesting the potent immunomodulatory nature of this promising group of HDPs. In LPS-induced inflammation, the capability of PAP to alleviate the production of IL-6 (and other mediators) was found to be achieved by the peptide’s decreasing effect on the secreted alkaline phosphatase (SEAP) reporter gene located downstream from the promoter of NF-κB, thereby inhibiting its activation and the subsequent release of pro-inflammatory cytokines [14].

IL-10, an anti-inflammatory cytokine originating mainly from macrophages and DCs [53], acts primarily as a suppressor of the TLR-agonist-induced production of different pro-inflammatory cytokines [54]. Therefore, IL-10 plays a key role in protecting the host from excessive inflammation and immunopathology [53]. In the present study, as a response to Poly I:C-induced inflammation, elevated production of the cytokine was observed, which was not influenced by the application of PAP. However, considering the IL-6/IL-10 ratio, the two higher concentrations of PAP exerted a decreasing effect on the elevation caused by Poly I:C. The IL-6/IL-10 ratio is increasingly referred to as a reliable marker of the inflammatory state [55] and has been observed to positively correlate with severe outcomes in patients with systemic inflammatory response [56] and neutrophil counts [57]. Consequently, besides their absolute concentrations, the relative levels of IL-6 and IL-10 might reflect the overall inflammatory milieu, showing the shift in the net balance between the pro- and anti-inflammatory cytokines [58]. Therefore, the decreasing effect of PAP on the ratio suggests the contribution of the HDP to the host’s anti-inflammatory efforts. In the present study, the strong positive correlations found between IL-6 and IL-10, as well as between IFN-γ and IL-10, also provide a useful insight into the immune response, suggesting the induction of IL-10 release driven by the pro-inflammatory mediators [58]. However, investigating further key elements of the downstream signaling pathways related to TLR2- and -3 could contribute to a better understanding of the overall inflammatory events, the lack of which is a limitation of our study.

As a response to different TLR-agonists and pro-inflammatory interleukins, the production of IL-8 by hepatocytes, macrophages, monocytes, and other immune cells is also stimulated, resulting in the recruitment of neutrophils, T cells, NK cells, and basophils, thereby stimulating inflammation [54]. In the present study, the highest concentration of PAP, alone and combined with Poly I:C, elicited a decreasing effect on the production of IL-8, indicating its contribution to alleviating inflammation. Even though there are no data available about the effects of papiliocins on IL-8, numerous insect-derived cationic HDPs have been observed to display the same decreasing effect [59–61]. Regarding LTA-induced inflammation, PAP was not able to restore the elevation of IL-8 release caused by LTA. Although the exact interaction between HDPs and LTA is an unanswered question yet, recent evidence suggests that certain cationic HDPs can show considerably high affinity to the anionic LTA molecule [39]. The resulting strong attachment might entrap the peptide, thereby lowering its local concentration near the cell membrane and inhibiting its direct effect [39]. On the other hand, this mechanism also “masks” the LTA’s binding sites necessary for evoking inflammation [39]. As it is suggested that HDPs that are likely to bind LPS tend to interact also with LTA [22], and PAP has been reported to show high affinity to LPS [14], it can be assumed that PAP might be entrapped by LTA in the present study also. As a consequence, the peptide could not reach a concentration high enough to exert its decreasing effect on the IL-8 levels, as PAP molecules were partly used up to bind LTA molecules. The same results found in the case of RANTES might also indicate a similar entrapment of PAP by LTA. Even so, the investigation of this event would have required further specific examinations, the lack of which is a limitation of our study.

RANTES, another inflammatory mediator examined in the present study, is produced by hepatic stellate cells, macrophages, and endothelial cells in the liver, contributing to the recruitment of peripheral macrophages and activation of tissue macrophages [62]. In addition, the chemokine promotes the polarization of macrophages to the M1 phenotype [62], which is responsible for the production of pro-inflammatory cytokines [19, 63], whereas the conversion to the anti-inflammatory M2 phenotype is inhibited by its action [19, 62, 63]. There has been increasing evidence suggesting the frequent involvement of RANTES in different liver diseases, describing it as a mediator of hepatic cell injury; however, its particular mechanism of action is not fully elucidated [62]. In the present study, both LTA- and Poly I:C-induced inflammatory conditions resulted in elevated levels of RANTES, the latter of which was alleviated by PAP at its highest concentration, suggesting its protective effect on the liver. To the best of the authors’ knowledge, this chemokine has never been investigated either in connection with PAP or any other derivatives of papiliocin. However, other HDPs of different origins were also described to decrease the production of RANTES in inflammatory stimuli, thereby alleviating inflammation [64–66].

Henceforth, the impact of PAP on the immune response was investigated by determining the changing in the levels of IFN-γ, a type II interferon synthesized by macrophages, DCs, and activated lymphocytes [67]. Initially, it was thought to have a key role primarily in the anti-viral response; however, it is now well-known that IFN-γ is crucial for protecting the host against a wide range of pathogens and inflammatory stimuli [68]. Nevertheless, its over-activation can lead to tissue damage, necrotic events, and immunopathology by promoting, among others, the production of other pro-inflammatory mediators, ROS, and reactive nitrogen species (RNS) [68]. In the present study, only Poly I:C-evoked inflammation resulted in an elevated level of IFN-γ, which increase was not significantly influenced by PAP. In contrast, according to bibliographic data, several other cationic HDPs of insect origin have been reported to affect the production of IFN-γ [37, 60, 69]. Still, it is of great importance to highlight that different HDPs display highly varied immunomodulatory activities, also depending on the specific experimental circumstances [7]. Therefore, the further investigation of PAP’s influence on the interferon-response involving type I interferons could provide useful insights to evaluate this result of the present study.

### Redox markers

In recent years, an increasing number of studies has revealed the cellular interplay between inflammatory and oxidative events, describing their mutual influence on each other [42, 70]; therefore, the present study aimed to determine the levels of EC H_2_O_2_ and Nrf2. This tightly regulated connection of the redox and immune state is suggested to befall, among others, through the interaction between the transcription factors NF-κB and Nrf2 [70]. Being able to conversely regulate the expression and activation of the two molecules, this crosstalk allows a highly coordinated immune response [42]. This interplay is suggested to be confirmed by the present study, as high or moderate positive correlations were found between all the measured inflammatory markers and at least one of the redox parameters. Regarding the levels of EC H_2_O_2_, the two highest concentrations of PAP were able to restore the elevation caused by Poly I:C, suggesting the antioxidant nature of the HDP. Even though the impact of papiliocins on the oxidative state has not been frequently examined, our findings are in agreement with the observations on the parent peptide papiliocin, which was able to decrease the intracellular H_2_O_2_ levels caused by oxidative stress in CaCo-2 human colorectal adenocarcinoma cell line [71].

In the case of Nrf2, LTA-induced inflammation contributed to an elevated level of the transcription factor, whereas in both inflammatory conditions, the addition of PAP resulted in a decrease. Under physiologic circumstances, Nrf2 is mainly degraded by Kelch-like ECH-associated protein 1 (Keap1) in the proteosome [72]. However, upon oxidative stress, Nrf2 escapes from the complex and translocates into the nucleus, thereby activating the gene expression of various cytoprotective proteins [72] and suppressing the pro-inflammatory ones [70]. As a result, Nrf2 is described as a major regulator of the antioxidant defense system [70, 72]. It has already been described that TLR2-agonists can act as activators of the Nrf2-signaling pathway, reflecting the cellular efforts to enhance the expression of antioxidant molecules and hence, support survival [70]. Based on these, the reduced levels after applying PAP in inflammatory conditions suggest that owing to the beneficial effects of the peptide on the immune response, the cells were not forced to augment the antioxidant processes anymore. The positive correlation found between EC H_2_O_2_ and Nrf2 also confirms this explanation, suggesting that the antioxidant system was activated according to the current oxidative state. Interestingly, when applied alone, PAP exerted a decreasing effect on Nrf2 concentration, moreover, IL-8 concentration was also decreased by the same treatment. Since it has been recorded that IL-8 can promote the compensatory elevation of the Nrf2 level [73, 74], and in the present study, the two parameters highly positively correlated with each other, it can be suggested that the reduced cytokine level contributed to the mild presence of Nrf2. Nevertheless, additional research is required to better explain these events. In addition, there are only a few studies available about the effects of HDPs on the Nrf2-signaling pathway, describing both the activation [75] and suppression [30] of the transcription factor in hepatic cells; therefore, their exact mechanism of action still remains unclear.

Taking the results together, PAP was found to influence the production of most of the measured parameters, indicating its potent immunomodulatory capability in inflammatory conditions; however, when applied alone, the peptide was not characteristically found to result in changes. These observations suggest that the treatment of PAP under physiological conditions is unlikely to interfere with immune processes, whereas, in the presence of inflammation, the HDP is able to exert immunomodulatory activity to protect the host, preferring to act towards the anti-inflammatory processes. Nonetheless, given the varying impacts of PAP on the inflammatory environments evoked by the different TLR-agonists used in this study, further investigation is required to better understand the peptide’s particular mechanism of action.

## Conclusion

The goal of the present study was to investigate the effects of PAP on the inflammatory response and oxidative state of a primary hepatocyte–non-parenchymal cell co-culture of chicken origin. Based on our results, PAP seemed to exert no cytotoxic effects on chicken hepatic cells, suggesting its safe application in poultry. Moreover, PAP displayed a robust modulatory activity on the immune response as it was able to decrease the levels of IL-6, IL-8, and RANTES, as well as the IL-6/IL-10 ratio. Therefore, the peptide is suggested to provide beneficial effects to the host in Poly I:C-triggered and LTA-induced inflammatory conditions. Furthermore, the examination of the activity of PAP on EC H_2_O_2_ and Nrf2 levels showed that the HDP might act as an antioxidant and promote the elimination of ROS. Based on our results, PAP possesses a highly potent immunomodulatory property, and it might be a promising candidate for replacing ABs, thereby contributing to the reduction of AMR in the future.

## Supporting information

S1 Table Showing correlation coefficients (r), p values and descriptions of correlations between the tested parameters. Correlations were described as “very high”, “high”, “moderate”, “low”, or “negligible”, based on the r value being ±0.90–1.00, ±0.70–0.90, ±0.50–0.70, ±0.30–0.50, and 0.00-±0.30, respectively.
(XLSX)
